# The Simple Treatment of Disease; Deduced from the Methods of Expectancy and Revulsion

**Published:** 1843-04

**Authors:** 


### ART. IX.

1. *The Simple Treatment of Disease; deduced from the methods of Expectancy and Revulsion.* By JAMES M. GULLY, M.D.—*London,* 1842. 12mo, pp. 128.

2. *Beiträge zur wissenschaftlichen Heilkunde.* Von Dr. C. A. W. RICHTER.—*Leipzic,* 1842. 8vo, pp. 271.

*Contributions to Scientific Medicine.* By Dr. C. A. W. RICHTER.—*Leipzic,* 1842.

THESE two works, though dissimilar in title, have a general correspondence in subject and object. We therefore class them together, under one head, for the sake of comparison. We shall, however, notice each separately.

I. Dr. Gully commences his work with an introduction in which we have a brief but clear and neat sketch of the medical theories that from time to time have given a tone to practice. In Chapter i. the expectant method of treatment is traced historically, and, as far as the limited space will allow, satisfactorily. It is thus defined:

"The expectant plan of treatment, strictly speaking, involves the necessity of awaiting the operations of nature in the case of disease. A more extended meaning includes the addition of artificial means, only as auxiliary to the efforts of nature, those efforts being the sole guide of the practitioner. It is in this latter sense that expectant medicine is to be understood. A physician, therefore, who professes to act upon expectant notions of treatment, takes it for granted that the constant tendency of the diseased body is towards cure, and this, for the most part, by the erection of certain modes of vital action in other parts of the frame than that which is morbid, and by the elimination of certain matters from the different emunctories. Thus he observes that the appearance of an eruption on the skin is simultaneous with the removal of some internal disorder; and that a fever usually declines just as the kidneys, the lower bowel, or the skin, pour out their respective excretions copiously. Acting on the hint thus afforded by the system, he waits for the commencement of any of these signals of relief, and then urges his remedies in the same direction that nature takes; not purging, if the kidneys are dissipating the fever, nor giving diuretics when the skin is bathed in sweat for a similar kind of purpose. This is the leading idea in his treatment. In the details, he follows it out by giving diluents plentifully, because nature craves for them, and forbids strong food, because nature loathes it; orders the recumbent posture, because the frame inclines to it; commands the withdrawal from food and light, because they are painful; and recommends abstraction of the mind from thoughts that require exertion, since he knows that nature is not capable of a genuine effort at such a time, but inclines rather to languor. He further regulates the temperature of the chamber with great precision: looks to the bedding and the positions in the bed of his patient; in short gives particular attention to the physical comforts that may add to the ease of his frame. Moreover, he has reference to the age, sex, temperament, and other circumstances of the individual; varies his means with the different periods of the disorder; assists the imperfect crisis of it, &c.

"Thus an enlightened system of expectancy does not signify an indolent awaiting of the event of the disease, leaving that event to the chances of the corporeal powers. It supposes a very precise knowledge of the history, particular characteristics, and progress of diseases; a close observation of their different periods, and the directions into which their crises may flow; and a very nice attention to the physical and moral regimen of the patient." (p. 32 et seq.)

This is a very fair account. Such doctrines are of course theoretical; they are not pure empiricism. The following sentence is not quite in accordance with what we think the fact:

"In diseases of a chronic character, which only involve the functions of a part, he attaches importance to negative rather than positive means; these consisting in the quiescence of the organs affected. He holds that if this be strictly enforced, the natural powers will always restore the healthy condition; and that, on the other hand, if these powers be taxed to carry on their morbid function in addition to the restorative process; or, further, if they be interrupted by imagined auxiliaries in the shape of medicines, such condition is postponed or hurried into the contrary extreme of organic disorder." (p. 37.)

There are many cases of chronic functional disease, in which the expectant physician sees only the commencement of structural disease; and in which the tendency of the natural powers is always towards greater disease and to death. In such cases he has no hope of curing, but is too happy if he can retard or arrest this tendency to a morbid state.

In the second chapter, the progress of the revulsive method is traced, and the remedial means most commonly used to carry it out, are detailed. This method is actively antiphlogistic and pharmaceutic. The abstraction of blood, purgatives, emetics, diuretics, diaphoretics, and counter-irritants, are the antiphlogistic means; expectorants, deobstruents, carminatives, and the like, the pharmaceutic.

"A physician acting on this treatment, in a case of simple fever, would, in the first place, attempt to 'cut it short,' as it is termed, by a copious bleeding from the arm, and, immediately on the limb being bound up, by an emetic. If this does not dispose of the fever in a few hours, he administers repeated doses of calomel, opium, and antimonial powder, with some powerful cathartics, in which he persists, in the expectation of causing healthy evacuations. In the meanwhile, time is found for the administration of a mixture of sudorifics and diuretics. So things go on for six or seven days, the patient being told, as regards diet, that he can take 'any little thing he fancies,' but not beef-steak or mutton-chop. At length the brain gives symptoms of being affected —delirium supervenes. Then begins the array of revulsives on the external surface: the head is shaved, ice is applied at the back and front, and a blister on the top of it. The delirium passing from the furious to the muttering kind, the tongue becoming dry and brown, the limbs utterly powerless; in short, all the signs of a typhoid state appearing, the stomach is once more tried with wine and other stimulants, the feet are blistered, and exhausted nature sinks; or if, in spite of all, the jaws of death are escaped, the body drained of its blood, worn out after the enormous stimulation, and utterly thrown out of the rhythm of its sympathies and functions, drags through a prolonged convalescence, with a tardy recruiting of the quantity, and still more slow restoration of the quality, of the vital fluid it had lost, and of the nervous energy it had previously possessed. Even in this state a little revulsion is essayed in the form of bitter tonics, carminatives, antispasmodics, &c., and sometimes a relapse into another species of fever called nervous is thereby brought about." (p. 66.)

Dr. Gully remarks that Hippocrates never practised the revulsive method before the fourth day; but does not notice the fact, that it was the peculiar doctrine of critical days that directed his practice, and the observation of which contributed so materially to his fame. The intermittent nature of disease must most certainly be better understood before we can practise medicine scientifically, whether we practise the revulsive or expectant plan. Dr. Holland has an interesting essay on the subject in his "Medical Notes and Reflections;" and more recently, Dr. Laycock has attempted to demonstrate a *general law* of vital periodicity; and to point out the measure of time by which the periods are regulated. If his researches prove to be correct, a considerable change must necessarily take place in both the theory and practice of medicine.

The third chapter is occupied with the simple treatment of disease, and constitutes the remaining half of the volume. We anticipated an elucidation of the merits peculiar to the two systems already reviewed, based upon modern discoveries in anatomy and physiology; or at least we expected a philosophical comparison of blind empiricism on the one hand, and of scientific hypotheses on the other, so that an eclectic method, simple from its grandeur, would be the result. We must, however, be honest, and say this third chapter has sadly disappointed us, for it has presented us with nothing more than a system of medicine of the author's own, and, what is curious, a system remarkable for its diminutive proportions. Dr. Gully slides into the statement of his own views very neatly.

"........ As in my opinion, a choice is to be made from, rather than between, both [methods], the comparison can only be established with reference to their propriety in the individual diseases of the body. This implies the statement of certain peculiarities in the vital actions, and of the relative importance of the various series of organs which minister to those actions in health and disease. Further, it will be requisite to enumerate some generally-received propositions regarding the sympathies, by virtue of which disease is propagated from one organ or series to other organs. After which it will not be difficult to point out the instances in which expectant and revulsive medication are respectively applicable." (p. 89.)

The sympathies mentioned play between the organs of vegetative and animal life, or between organs of each class. "Irritation of the stomach, for instance [the stomach is a vegetative organ], begets what is called sick headach," or an affection of an animal organ—the brain; or "disordered stomach begets cough with expectoration or sore throat;" in other words, disorder of a vegetative organ "begets" diseases of vegetative organs. Diseases vary as they commence in organs of vegetable or animal life, and require proportionately different treatment. The grand truth, however, is "that *no one sinks under disease until it has invaded the viscera of vegetative and animal life.*" It would perhaps be the fairest to Dr. Gully to quote his own "*resumè.*"

"Disease commences on the external surface or skin; the internal surface or viscera of vegetative life; or in the brain and spinal cord, the viscera of animal life. Commencing on the external surface, it is propagated to the internal and to the brain. Commencing in the internal surface, it is propagated to the external and to the brain. Or in the brain, to the viscera of vegetative life and the skin. So long as disease commencing in the skin is not propagated beyond a certain degree to the viscera, life is not compromised. So long as disease commencing in the viscera of the abdomen and chest is not propagated beyond a certain degree to the brain, life is not compromised. So long as disease commencing in or propagated to the brain does not react on the other viscera, beyond a certain degree, life is not compromised. Hence the axiom that 'death comes only by the viscera.' The great aim of treatment, therefore, is to withdraw irritations from the viscera, and to save animal life. The dependence of the abdominal viscera upon, and their connexion with, the brain, is not so great and immediate as those of the chest. General febrile states, whether owing to inflammation or irritation of the abdominal mucous lining or not, certainly begin and disappear with them. The same applies to chronic diseases of that membrane. In preventing the propagation of these to the brain, the expectant treatment is applicable, for the reason given in the last paragraph. In the acute disorders of the coverings of the abdominal viscera, the propagation to the brain being more speedy, revulsion is more demanded. The dependence of the thoracic viscera upon and their connexion with the brain, is more immediate than those of the abdomen. Revulsion on the latter is therefore necessary and proper in acute diseases of the chest. In its chronic disorders, revulsion on the external surface is more decidedly indicated. Disease of the brain invariably operates on the viscera of the abdomen and chest. If acute, it requires active revulsion both on the external and internal surfaces. If chronic, the viscera of vegetative life becoming deeply affected and reacting upon the brain, revulsion on them is not so applicable as on the external surface.

"In all disease, the end of treatment is to spare the viscera. When the abdominal viscera are prominently disordered, they should be spared at the expense of the skin. When the viscera of the chest and brain are the seats of disorder, they should be spared at the expense of the digestive organs and skin." (p. 189 et seq.)

Expectancy is passive treatment, revulsion active treatment. We must draw the sword of revulsion on diseases situate in organs of animal life, for the citadel of life is in danger; we must let vegetative organs alone, and their diseases will disappear spontaneously; or, if they threaten to spread to organs of animal life, we must starve or poultice them into submission. They must not be irritated by medicines. Senna is too powerful a cathartic; a teaspoonful of castor-oil with four or five grains of rhubarb is quite enough; but better than both, is a purgative enema to "cleanse out the lower bowel."

The treatment of acute and chronic indigestion, of fevers, of diseases of the thorax and brain, is then laid down. We have room for a case of inflammatory fever only. The patient was a gentleman, twenty-nine years of age, and fond of dining out and going to balls.

"In this case there were the usual signs of inflammatory fever: headach, suffused eyes, dry tongue, excessive thirst, hard, rapid pulse, torpid bowels, scanty urine, dry, hot skin, prostration of strength though the body was restless, sleeplessness, &c. The abdomen was sensitive on pressure, although to no great extent. The patient being in bed, the room rendered obscure, and a portion of the windows opened to admit air, an enema of castor-oil and warm gruel was administered, which caused an evacuation of hard and somewhat dark-coloured fæces. For the double purpose of quenching thirst, and acting upon the upper portion of the canal, small and frequently repeated doses of carbonate of potass, with citric acid taken in an effervescing state were given. As soon, however, as the enema had operated, and after two or three doses of the effervescing medicine, fomentation of the abdomen with flannels wrung out of boiling water, and changed every five minutes, was assiduously practised during three hours. Two hours after this had been suspended the saline acted rather freely, and was discontinued for a few hours. Meanwhile, cold water and soda-water were permitted in quantities of two wine-glasses at once, and at intervals of twenty minutes or half an hour. The forehead and temples were wetted with Cologne water, the evaporation of which was constantly encouraged by fanning. The face, hands, and feet were sponged with tepid water, and carefully wiped, every two or three hours. As far as it was practicable, all noise was withdrawn, and the visits and conversation of friends forbidden. In the evening, when, as is usual, the febrile heat and restlessness somewhat augmented, the fomentation and saline draughts were recommenced, the former continued for three hours again, and the latter during the greater portion of the night." (p. 122.)

Well, this kind of treatment "was continued for eight days, with some slight variations as to the time and mode of evacuating the bowels, as to the length of the fomentations, and as to the fluids to be taken, which consisted sometimes of rice-water, barley-water, and occasionally of lemonade," and the patient was able to walk about his room in twenty days from the commencement of the attack.

Another case illustrates the treatment of "typhoid fever," but the details are similar except in some minor matters; as, for example, flour of mustard is used for fomentation, and a linseed poultice applied to the abdomen. Next comes a case of "fever with rheumatic pain," then one of infantile remittent. The treatment of the exanthemata occupies no more than three widely printed duodecimo pages,—so simple is Dr. Gully's method.

The treatment of diseases of the lungs, heart, and brain follows; the principles of Dr. Gully, so far as we can see, differing little from the principles of any other tolerably well-informed practitioner, provided the theoretical phrases of our author be translated into common nosological language. Dr. Gully, we opine, has not had an extensive practice, at least among the middle and lower classes of society; or, he can never mean that such "simple treatment" as we have detailed can be generally applicable in such a sphere of life. Where is the Cologne water to come from? and the relays of nurses to foment, and the fanners, the ice, the thermometers to regulate temperature, the inhalers? These probably would be within the means of one patient in five hundred, and not one nurse in twenty would attend to the directions.

In chronic diseases of the lungs Dr. Gully recommends the use of opiate liniments, and we join him in that recommendation. In a note, Dr. Gully states that metastasis to the heart and brain in "rheumatic inflammation of the limbs" is much less frequent than commonly supposed. He has treated, or seen treated, "a vast number of cases," and can only testify to having witnessed seven or eight. Dr. Gully ought to know, that if nobody will doubt his statement, nobody will attach any weight to it. Where is the record of this "vast number?" Dr. Gully thinks it a newly discovered fact that atonic congestion of the brain may be brought on by profuse bloodletting; we thought the fact was well known to all but those illiterate practitioners who pride themselves upon never reading a book, and assume the title of "practical men."

We believe we have given a fair view of the nature and contents of Dr. Gully's publication, and our readers will be fully able to estimate the value of it for themselves. We suspect it will not answer the author's purpose. It is neither popular nor scientific. The literary execution of the work, excepting in the earlier pages, is very indifferent; it has been somewhat of a penance to us to read it. Technical words are continually used with a canting or popular meaning. A patient suffering from "typhoid fever" sometimes took ice into his mouth instead of iced water; and we are told that "the *gusto* with which he crushed it between his teeth was highly illustrative of the *intense fever* of the stomach." The same patient had a linseed-meal poultice applied "over the *whole body*," and the ludicrous idea is excited of a patient being put to bed after being dipped to the ears in a tub of the cataplasm. "Body" means abdomen with Dr. Gully; a clinical use of the word we always thought peculiar to ladies and nurses. The physiology displayed in the volume is far from precise, and not seldom gratuitous.

II. The work of Dr. Richter comprises three sections. The first of these is an introduction to the other two, and consists of an exposition of the principles which should guide physicians in their researches. The second treats of the spontaneous origin and cure of disease. The third considers the origin and cure of disease by art. The whole may be viewed as an attempt to connect the modern discoveries in physiology and pathology with empirical theories in medicine.

Hippocrates first stated the fundamental doctrine of the expectant method, namely, that the constant tendency of the diseased body is towards cure. This tendency, our author shows, is dependent upon the general laws of development and of self-preservation in the organism; a view already fully expounded in a previous publication of his on the "water-cure," but applied in the present work to general pathology and therapeutics.

The general idea respecting the so-called *vis medicatrix naturæ* is this: that the organism and the disease are conflicting powers, and that the *vis medicatrix* steps in as a third power, to the assistance of the organism. The therapeutics founded upon this idea consist, mainly, in an idle waiting for the result of the conflict, being a purely *methodus exspectativa.* Our author acknowledges, indeed asserts, that there is a *vis medicatrix naturæ;* but maintains that it is not (to use the expression of Van Helmont) an *ignotus hospes* in the system. It is no other than the innate organizing force of the organism, of which the *nisus formativus* of Blumenbach is a modification, not only evolving the individual from the ovum, but carrying him on to his full development, and maintaining him in existence. In fulfilling the latter duty, there is a continual alternation of life and death in the system. This power both organizes unorganized matter and disorganizes vitalized matter; separating the latter also from the system, after it has fulfilled its uses in the organism, and has become (if detained) a hurtful agent. This separation is the act of the so-called *vis medicatrix.* If the recomposing and decomposing processes are in equilibrium there is health; but if the effete matter be not cast off, or a hurtful agent enter the system from without, the equilibrium is destroyed, and the innate vital force sets up an action to restore it (therapia interna), the resulting phenomena being the phenomena of disease. So that morbid action consists in an interruption of the renewing or reformative process concurrently with alterations in the qualities of the blood, and a reaction of the innate vital force to restore the normal state. The *schädliche Potenz*, or hurtful agent, is eliminated from the system through the secreting and excreting organs; or, not being fully eliminated, is localized in some one or more special structures, thus giving rise to various constitutional and chronic local diseases. According to these views, healthy and morbid action are identical; or rather, the healthy is the type of the morbid.

Setting out from these principles, Dr. Richter examines the pathology of acute and chronic diseases, explaining their diversities and similarities, and deducing many very ingenious, and, we must say, somewhat paradoxical propositions. Fever (even hectic fever) is a healthy process. The innate vital force, feeling the presence of a hurtful agent in the system, attempts its removal after the same manner as it removes effete matter. The decomposing process overbalances the recomposing, or renewing; a colliquescence or colliquation of the various organs is the result; and this continues until the object is attained; when the recomposing process assumes the superiority, until the equilibrium between the two processes is reestablished. If the hurtful agent is not eliminated it is located in an organ, and the innate vital force attempts its removal by colliquation of that organ, giving rise to the various changes of structure described by pathological anatomist, and to the general symptoms of local disease. Hectic fever is in reality a healing process, set up to expel the hurtful power from the organ to which it has retreated; and is injurious only, because the hurtful power is of such a nature, or so situate, as to require for its expulsion a greater effort than the vital machinery will bear; so that the recomposing process is never reestablished, and the colliquation goes on until dissolution of the organism, or death takes place.

As the hurtful powers differ in composition, so they have varying affinities for the different organs and structures of the system. The poison of rabies, as well as mercury, passes off by the salivary glands. Some hurtful agents fix more particularly in the osseous structures, others are eliminated from the skin and mucous membranes; and Dr. Richter asserts that the globules of mucus present under the microscope differences in form, colour, and size, as diseases differ. The results of his researches on this part of the subject, are promised to the public at a future opportunity. Dr. Richter is opposed to the opinion of Henle respecting the existence of a *contagium animatum* as a cause of disease, but draws a very curious parallel between the fermentative process and the development and increase of contagious matter, especially in the exanthemata. The idea of a *contagium animatum* is by no means novel; and the new views respecting the vegetable composition of yeast, promulgated by Caingard de la Tour and Schwann, and confirmed by Dr. Richter, may be considered rather favorable than otherwise to Henle's notions. According to the new views referred to, yeast is composed of organized cells capable of reproduction, like the simplest cryptogamia, and we do not see why the parallel drawn by Dr. Richter between the phenomena of fermentation and of the exanthemata, may not be extended so as to comprise the first cause of both. Without saying one word in favour of Henle's views, we may fairly assert that the objections of Dr. Richter are peculiarly invalid. A *contagium animatum* is at least as probable as a fermentation of humours.

We cannot follow Dr. Richter through his pathological details. The treatment necessarily flowing from the principles he advocates, is purely eclectic. Health and disease exist, and disappear, as the action of the principle of self-preservation in the organism takes place under homogeneous and friendly, or heterogeneous and unfriendly, external influences. In treating disease, the sagacious physician will act in accordance with these views. When the innate vital force is equal to the restorative effort, he will wait the result; or, in other words, adopt the expectant method. If its action is blindly violent, and likely to lead to serious local disease, or to dissolution, the treatment will be revulsive. If it wants assistance, the physician will consider by what organs, and in what mode, it is attempting to expel the hurtful power, and direct his remedial means accordingly. He will remember the maxim, "*ubi irritatio, ibi fluxus*," and that observation has demonstrated, not only that the various hurtful powers have varying affinities for different organs, but that remedial agents have like varying affinities. Mercury excites abnormal plasticity in mucous membranes; copaiba developes a morbillary eruption; belladonna a scarlet rash. Narcotics induce a thickening and swelling of the nerves;—the aqua lauro cerasi, for example, so changes the pneumogastric nerve. Strychnia has distinctly chemical relations to the organized substratum of the spinal cord; quinine evidently acts upon the ganglionic system. Watching, therefore, the efforts of the innate vital force, and the symptoms it excites, the sagacious physician will assist it, by setting up a mode of action analogous to its own; treating the disease, in fact, on the homœopathic maxim of *similia similibus curantur;* for as morbid action is healthy action, the cure must be the same as the disease,—medicines only assisting the healthy action of nature in throwing off the hurtful power,—the *schädliche Potenz.* The application of this maxim of homœopathy must, however, be limited within the principles Dr. Richter lays down. Thirst, for example, must not be cured by inducing thirst. This sensation is excited by the innate force, that the free imbibition of water may be provoked. Water promotes the colliquative, or decomposing process; and thirst is most observed in fevers in which an active colliquative process is most required to get rid of the hurtful agent.

In short, Dr. Richter is a humoral pathologist, and an eclectic practitioner. He recommends all and every method of treatment,—the expectant, revulsive, simple, homœopathic, hydriatic. This is nothing new. The judicious and eclectic physician has long acted upon the views of Dr. Richter. Common sense informs us that each method may be useful in suitable cases; and common sense equally assures us that none but quacks and foolish enthusiasts will exclusively apply any one of them to all cases indiscriminately. The diplomatized Hydriaticians are just as quackish and dangerous as the untitled Morrisonian revulsionists; perhaps more dangerous, certainly more quackish.

The result of the comparison of the two works before us is unfavorable to Dr. Gully. Dr. Richter's is purely scientific, and breathes the true spirit of a philosopher; Dr. Gully's exhibits on the part of its author the tricky wish to stand well with the profession, and catch, at the same time, the popular ear. Dr. Gully will find out that, in this respect, he has attempted too much.

---

